# A Case of Primary Sternomanubrial Osteomyelitis With Oxacillin-Resistant Staphylococcus aureus (ORSA) Bacteremia

**DOI:** 10.7759/cureus.25313

**Published:** 2022-05-25

**Authors:** Marc Assaad, Rachelle Hamadi, Khalil El Gharib, Gennifer Wahbah Makhoul, Neville Mobarakai

**Affiliations:** 1 Internal Medicine, Staten Island University Hospital, Staten Island, USA; 2 Infectious Disease, Staten Island University Hospital, Staten Island, USA

**Keywords:** chest wall pain, sternum, methicillin resistant staphylococcus aureus (mrsa), sternoclavicular joint (scj) septic arthritis, osteomyelitis

## Abstract

We herein report the case of a previously healthy 26-year-old male patient who presented to our hospital with chest pain and fevers. Investigations revealed oxacillin-resistant *Staphylococcus aureus* (ORSA) osteomyelitis of the manubrium, for which no inciting event or background was identified, classifying it as primary sternomanubrial osteomyelitis (PSO). The patient was appropriately treated with intravenous antibiotics, resulting in clinical improvement. The sternomanubrial site without trauma has rarely been described in the literature.

## Introduction

Primary sternomanubrial osteomyelitis (PSO) is an unusual entity in healthy adults and sternal osteomyelitis represents 0.3% among all cases of osteomyelitis (OM) [1-3]. Manubrial osteomyelitis has been described once in the literature, was secondary to tuberculosis and complicated by abscess formation [4]. While sternal osteomyelitis can be primary or secondary, PSO most often results from hematogenous spread [1,5] and is an uncommon presentation in previously healthy adults [1]. The most common type of sternal OM is secondary, and is usually a complication of sternotomy, chest trauma, cardiopulmonary resuscitation or subclavian catheterization [1,5].

Associated risk factors for PSO are human immunodeficiency virus (HIV), intravenous (IV) drug use, diabetes mellites (DM) and liver cirrhosis [1,5]. We report the case of a 26-year-old healthy patient who presented to our medical center with an acute sternomanubrial OM.

## Case presentation

This is the case of a 26-year-old healthy male, with no prior medical history, who presented to the emergency department for evaluation of acute left-sided chest and shoulder pain of two days duration. Pain was located mainly at the left pectoralis muscle area along with the left clavicular bone and is described as acute, stabbing, and radiating to the left shoulder and the left subscapular area. A day after the pain onset, patient developed fever, myalgia, and chills. He denied intravenous drug use and any blunt or penetrating trauma to the chest. Patient had immigrated from Peru to the United States of America three months prior to his presentation. In the US, patient was unemployed and did not participate in any physical activity.

On admission, patient was febrile to 103.3 F and his physical exam was remarkable for neck stiffness and chest wall tenderness, most prominent along the left pectoralis muscle, left clavicle and left shoulder. There was no tenderness along the left sternoclavicular joint. Patient did not have any erythema, edema, or crepitus at these sites. Physical exam failed to reveal any track marks suggestive of IV drug use. Laboratory workup was remarkable for leukocytosis 14400 /uL with neutrophilic predominance 94%, serum lactate dehydrogenase of 276 U/L, creatinine kinase of 469 U/L, and normal troponin level. Erythrocyte sedimentation rate (ESR) was 17 mm/hour however C-reactive protein (CRP) was markedly high at 198 mg/L. Glycosylated hemoglobin was 5.6 and rapid HIV antigen/antibody test was negative. Electrocardiogram did not reveal any ischemic changes and an urgent computed tomography (CT) scan of the chest with IV contrast showed mild left basilar atelectasis and was negative for chest wall abscess, necrotizing fasciitis of pectoral muscle, and pulmonary embolism. CT scan was also negative for left clavicular, sternoclavicular and sternocleidomastoid joint involvement. Patient was started on broad-spectrum antibiotic therapy including vancomycin.

**Figure 1:**
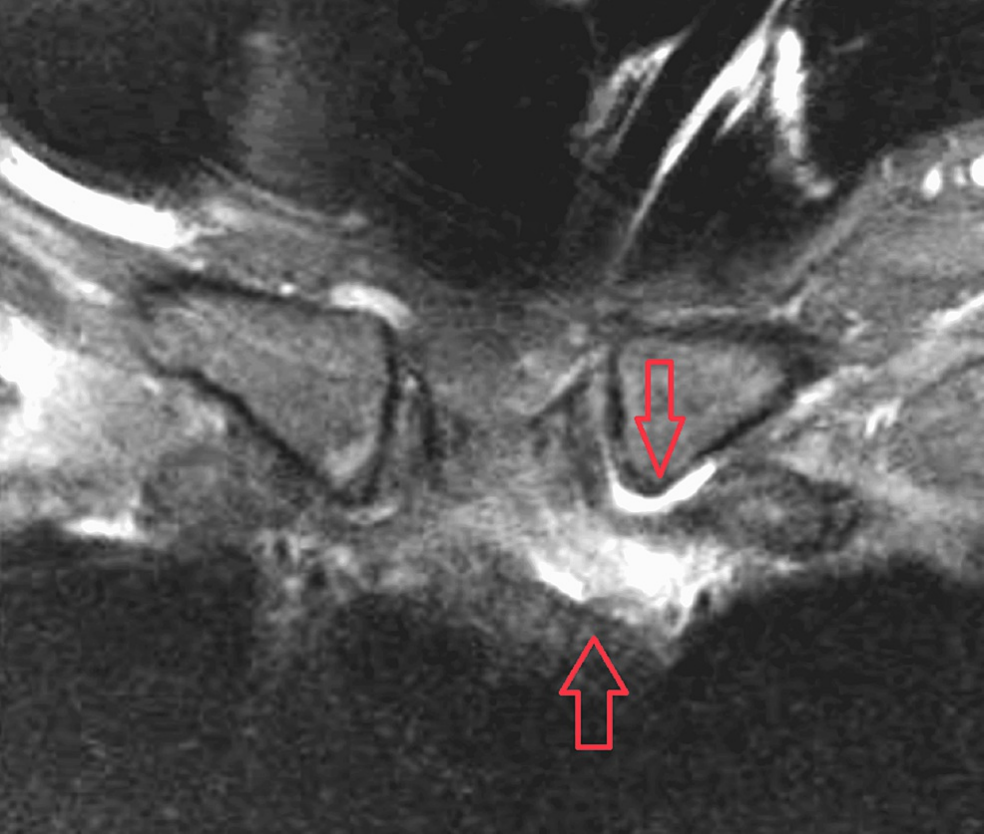
Magnetic resonance imaging of the chest showing osteomyelitis in the manubrium (arrow) with associated phlegmonous change and evidence of septic arthritis (arrow) in the left sternoclavicular joint

**Figure 2:**
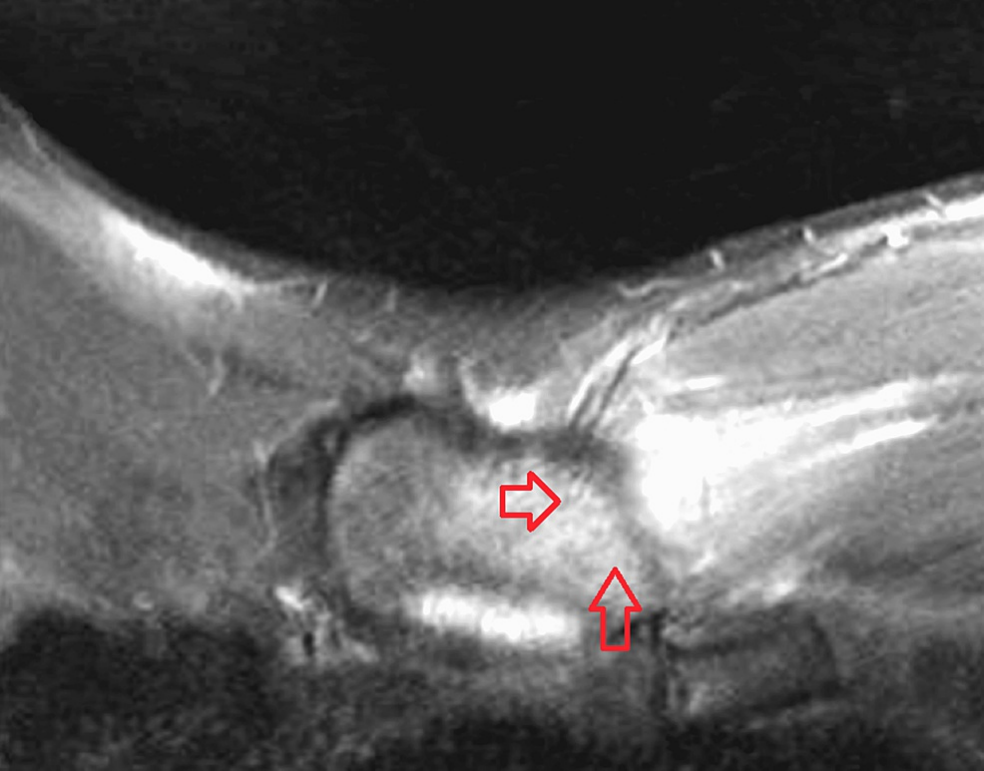
Magnetic resonance imaging of the chest showing osteomyelitis in the manubrium with associated phlegmonous change (arrow)

Blood cultures (four bottles) obtained on admission were positive for oxacillin-resistant *Staphylococcus aureus* (ORSA) prompting the investigation for an underlying source. Transthoracic and transesophageal echocardiogram revealed no valvular vegetations. Urine drug screen was also negative for substance abuse. Left pectoral muscle fasciitis was suspected on initial presentation, and magnetic resonance imaging (MRI) of the chest, neck, and shoulder was performed. MRI revealed the presence of sternomanubrial osteomyelitis and septic arthritis of the sternoclavicular joint with associated phlegmonous change (Figures 1, 2). No pathology was found in the left pectoralis muscle, left clavicle, left shoulder or scapular area. The left sternoclavicular junction did not appear to be infected clinically and an orthopedic evaluation had similar impression with no need for a deep arthrocentesis.

Blood cultures became negative 48 hours on therapy and apyrexia was achieved within 48 hours. Hospital course was remarkable for the resolution of the left pectoralis and shoulder pain within two days, with the persistence of the subscapular pain during the first week of diagnosis. The patient was discharged home on six weeks of intravenous vancomycin from the day of negative blood culture.

## Discussion

Sternomanubrial osteomyelitis (SO) refers to an uncommon infection localized at the sternomanubrial joint. The sternomanubrial joint is a symphysis, a connection between the sternal body and the manubrium through the ossification of fibrocartilage with age. There are few reported cases of arthritis such as osteoarthritis or septic arthritis of this joint [6]. The manubrium is the attachment point of multiple muscles such as: pectoralis major, sternocleidomastoid, sternohyoid and sternothyroid muscle. Given the location of these muscles along with their neuronal innervation, referred shoulder and neck pain is commonly seen with SO: these muscles are innervated by the accessory nerve. An injury to the accessory nerve can cause dull shoulder pain [7]. OM of this area is further described as being of primary if no eliciting cause was identified besides the bacteremia, or secondary origin.

PSO is an unusual entity and is generally associated with multiple risk factors including immunodeficiency, subclavian vein catheterization, and IV drug use. There are few reported cases of *Staphylococcus aureus* bacteremia in which no risk factors are present in an immunocompetent individual. In these cases, it is presumed that a state of transient bacteremia leads to an infection of an arthritic site leading to subsequent septic arthritis [8,9]. The most common pathogen in PSO is *S. aureus*, but other bacteria have been implicated such as *Pseudomonas aeruginosa* in IV drug users [1-5].

The above report describes the case of a 26-year-old male patient with no significant past medical history or risk factors diagnosed with PSO and ORSA bacteremia. Thorough workup for another possible focus of the ORSA bacteremia in our patient remained elusive with the only significant finding being a chest MRI suggestive of a sternomanubrial osteomyelitis and septic arthritis of the sternoclavicular joint. The lack of symptoms and lack of exam findings at the sternoclavicular joint, the MRI findings were deemed unremarkable of significant disease by orthopedic surgery. The absence of a different focus of infection is significant in the diagnosis of PSO in our patient.

The workup for PSO is similar to any other osteomyelitis relying heavily on imaging and inflammatory markers, whereas treatment is based on a prolonged antibiotic course. Non-invasive diagnostic imaging like CT or MRI is helpful to rule out associated abscesses or septic arthritis which could require a more invasive diagnostic approach or therapeutic drainage. The treatment of PSO remains source control with prolonged antibiotic therapy for six weeks with possible ESR and CRP monitoring to guide the duration of antibiotics. Surgical debridement is limited to certain indications such as removal of necrotic debris, abscess, or presence of foreign body [1,3,10]. Some complications reported in the literature include formation of neck abscess, chest wall involvement, and extension of the disease into the mediastinum or the pleural space [10].

## Conclusions

The importance of this case resides in highlighting the possibility of sternomanubrial site of sternal infection besides the commoner sternoclavicular area. Pain in the scapula and *Staphylococcus aureus* bacteremia should include a thorough exam of the sternum as a possible source. The increased prevalence of ORSA in society, especially in the healthy population with no prior risk factors, should also raise our concern about antibiotic stewardship and the inappropriate use of antibiotics.
